# “Track style” children's fundamental movement skills test: construction and verification of an efficient evaluation system

**DOI:** 10.3389/fpubh.2024.1437473

**Published:** 2024-08-19

**Authors:** Yubo Wang, Shuang Liang, Baofeng Zhang, Lingyue Meng, Yan Xiong

**Affiliations:** ^1^Physical Education and Sports School, Soochow University, Suzhou, Jiangsu, China; ^2^High School to Nanjing Normal University, Nanjing, Jiangsu, China; ^3^Physical Education and Sports School, Guangzhou University, Guangzhou, Guangdong, China

**Keywords:** motor development, child, fundamental movement skills, assessment system construction, physical activities, Delphi method

## Abstract

**Objectives:**

This study aimed to develop an efficient tool for assessing children's fundamental motor skills, the “Track style” Children's Fundamental Movement Skills Test (TCFMST), based on theories of motor development integrated with Chinese cultural context and physical education teaching situations.

**Methods:**

Starting from a literature analysis, the study selected items from existing fundamental movement skill (FMS) assessments, textbooks, physical education and health standards, and children's movement guidelines to construct a pool of test items. Subsequently, the items were screened and optimized using the Delphi method. Finally, the feasibility, discrimination, difficulty, reliability, and validity of the constructed test were examined using testing methods.

**Results:**

The TCFMST includes three dimensions: locomotive skills, body control skills, and manipulative skills, with a total of 10 items. The difficulty and discrimination of each item are appropriate; the correlation coefficients for retest reliability range from 0.789 to 0.943 (*p* < 0.01). The results of exploratory factor analysis indicate that the common factors align with the hypothesized three dimensions, indicating good structural validity of the test. The concurrent validity results show a correlation coefficient of −0.510 (*p* < 0.01) between the TCFMST and the total score of TGMD-3, indicating a moderate correlation between the two tests.

**Conclusion:**

The TCFMST developed in this study has good difficulty, discrimination, reliability, and validity. It also features strong operability, a short duration, and high interest. It can serve as an important tool for monitoring children's fundamental motor skill levels.

## Introduction

Physical activity is indispensable for the health of children and adolescents, with extensive research (1) demonstrating its benefits for cardiovascular and metabolic health, as well as for enhancing physical and mental wellbeing. It is particularly crucial for weight management, a significant issue for today's youth (2). However, the surge in sedentary behavior (SB) is a growing concern, with strong correlations to increased obesity rates, adverse cardiovascular effects, and poor sleep quality (3, 4). Conversely, an increase in SB has been associated with elevated obesity rates, detrimental effects on cardiovascular metabolism, and reduced sleep duration, among other adverse health outcomes (2–4). FMSs serve as the innate framework for movement learning and are essential for the execution of complex physical activities and sports (3). The cultivation of FMS is not only the foundation for children's participation in physical activities (5, 6) but also underpins their future adaptation to various sports and sporting environments. According to the action development peak model proposed by Clark and Metcalfe, human motor development is shaped by a dynamic interplay between the individual and their environment, resulting in a process of continuous growth and change (7). Mastery of FMS during childhood predicts the level of physical activity in daily life in adulthood (8), and children who have better proficiency in these skills are more likely to engage willingly in sports and maintain an active lifestyle (9).

Since the first introduction of the “Fundamental Gross Motor Skills Test” by the American Physical Education Association (APEA) in 1924 (10), the development of the FMS test has undergone several phases: the initial formation period, the application review period, and the integration into the physical education period. The current characteristics of FMS assessments are as follows: (1) The purpose of assessment has shifted from screening for motor delays to promoting physical literacy. Initially, FMS assessments were targeted at special populations with cerebral palsy or delayed motor development (11), aiming at screening and diagnosis. In the current stage, however, the primary objective of assessments is to evaluate the development of FMS in ordinary children (12). (2) There is a coexistence of single and multiple evaluation methods (13). The current methods of assessment include outcome-oriented evaluations, process-oriented evaluations, and a combination of both process and outcome approaches. (3) The establishment of norms is more closely aligned with the cultural backgrounds of different countries. Accurate assessment and timely monitoring of children's FMS development are crucial for enhancing their health outcomes.

Newell's constraints model posits that motor development is influenced by the interaction between the individual, the environment, and the specific tasks at hand (14, 15). Given that children spend a significant amount of time in schools during the period of rapid FMS development, the assessment of children's FMS has increasingly come to rely on educational institutions. Consequently, contemporary assessment tools are being deeply integrated into the physical education contexts of various countries (16), manifesting in several key aspects: (1) The assessors are transitioning from rehabilitation specialists or child health professionals to physical education teachers, with more teachers being involved in the development of assessment tools; (2) The content of assessments is aligning more closely with national physical education curricula or standards, with test items often selected from common or traditional sports within each country; (3) The implementation of tests takes into account the physical facilities, equipment, and time constraints of schools and their physical education teachers.

It is important to note that due to differences in ethnicity, cultural environments, and physical education backgrounds among different countries and regions, nations are actively developing FMS assessments that are suited to their local physical education contexts (17). Consequently, internationally mainstream assessments may not be suitable for the Chinese physical education situation and school sports environment. This study, grounded in theories of motor development, takes into account the Chinese educational and cultural background, as well as the school sports environment, emphasizing the feasibility of factors such as time, space, equipment, and teachers' knowledge background in school sports practice. The study aims to develop the TCFMST and to standardize and validate it.

## Methods

### Study design

According to the classical test theory (CTT) (18), this study is divided into two main phases. The first phase involves constructing the TCFMST system through the Delphi method; the second phase is the validation of the developed TCFMST system for feasibility, reliability, and validity. The TCFMST items were sourced from motor development theory, existing assessments (19–30), and textbooks, with consideration for practicality in school physical education and student engagement (31, 32). This resulted in a bank of 41 items, including body control, manipulative, and locomotive skills (33).

Child motor development specialists, early childhood physical education experts, and seasoned primary and secondary school PE teachers used the Delphi method to refine the test, ensuring each item's importance, suitability, and operational feasibility (34). After two expert consultation rounds, the test was administered to 1,005 children in Suzhou, aged 7 to 8, to assess item difficulty and discrimination and to validate reliability and validity using retest reliability, construct validity (35), and concurrent validity, as the test's “track style” timing evaluation method precludes other reliability measures (36).

**Figure 1:**
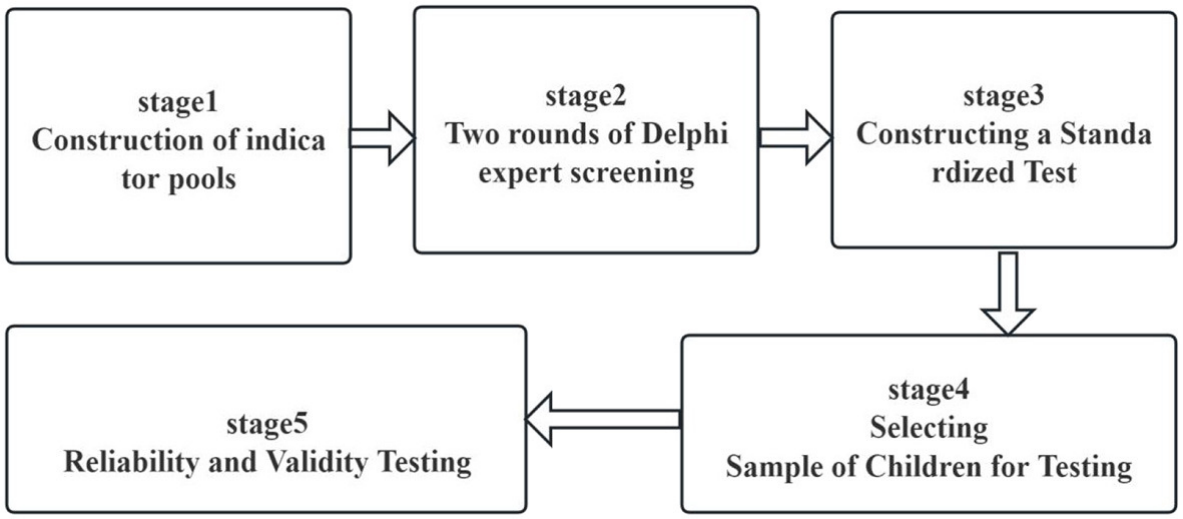
Study design.

Following analysis, the items were revised or removed to improve the TCFMST's scientific rigor. The study design is shown in Figure 1.

### Delphi method

#### Selecting consulting experts

The criteria for selecting experts include the following aspects: (1) experienced scholars and experts in the fields of child motor development and motor assessment; (2) scholars and experts engaged in school physical education, sports education training, and sports human science; (3) sports research and teaching staff or frontline physical education teachers with professional knowledge in child motor development and physical education, with a work experience of more than 10 years (37)^.^ A total of 14 experts were selected to participate in the consultation, and the basic composition of the consulting experts is detailed in Supplementary Table S1.

#### Construction of indicator pools

The dimensions of the TCFMST were primarily established based on Gallahue's framework, which includes three dimensions: locomotive skills, manipulative skills, and body control skills (31). The specific test movements were derived from three main sources: (1) literature on motor skills and motor development (32), (2) existing fundamental motor skills assessment tools (19–30), and (3) textbooks and children's physical activity guidelines. After careful screening, a preliminary set of test items was formed, comprising 10 body control skills, 11 manipulative skills, and 20 locomotive skills.

#### Design and distribution of expert questionnaires

The initial TCFMST was refined into an expert questionnaire. The first round of the expert consultation questionnaire included basic information about the experts, the TCFMST project consultation table, and a section for the experts to indicate their familiarity and judgment criteria for the test items. Additionally, the questionnaire provided detailed information about the test site, equipment, and the specific sources of the test items. The first round of expert questionnaires was distributed on 15 September 2023, and the collection was completed by 1 October 2023. A total of 14 expert questionnaires were distributed, and 14 were returned, achieving a recovery rate of 100%.

The second round of the expert consultation questionnaire maintained the TCFMST project consultation table and the section for familiarity and judgment criteria, while also presenting the average scores (M), standard deviations (SDs), and coefficient of variation for each test item from the first round. The second round of expert questionnaires was distributed on 15 October 2023, and the collection was completed by 5 November 2023. Out of 14 questionnaires distributed, 12 were returned, resulting in a recovery rate of 85.71%.

The questionnaires were distributed in person, via email, and by mail. Each questionnaire was individually distributed by the researchers to ensure that the distribution was uniform, controlled, and anonymous, adhering to the principles of repeatability, controlled feedback, and anonymity of the experts (38).

### Measure method

After two rounds of expert consultation, the test has been refined, but its quality cannot be guaranteed to reach the predetermined level. Therefore, it is necessary to conduct tests on the subjects. This involves calculating the statistical data for each item of the test from actual data and analyzing it to determine whether the difficulty and discrimination of the test items are appropriate. This analysis also aims to validate the reliability and validity of the test items (39).

### Subject

A convenience sampling method was used to select 1,100 children aged 7 to 8 years from six primary schools in Soochow city, specifically from grades 1 and 2. The selected test subjects met the following criteria: (1) they were in good health with no significant medical history that would affect their participation in physical exercise; (2) they had no moderate to severe cognitive impairments or developmental delays (as confirmed by parents or guardians); (3) they had obtained informed consent from their homeroom teachers and had written informed consent forms; and (4) written informed consent was obtained from all the participant's parents or guardians on a voluntary basis, indicating their understanding and agreement with the purpose and procedures of the study. However, 95 subjects were excluded due to reasons such as absenteeism, leaving a total of 1,005 effective subjects (Table 1). The research protocol for this study was approved by the Ethics Committee of Soochow University (review number: SUDA20240129H02).

**Table 1:** Demographic characteristics of the child participants.

	**Gender**	**n**	**Age (M ±SD)**
7.0	Male	243	7.43 ± 0.35
	Female	247	7.39 ± 0.37
8.0	Male	261	8.51 ± 0.29
	Female	254	8.48 ± 0.30

### Test content

#### TCFMST

The testing locations were outdoor basketball courts and indoor track and field areas within the schools. The test administrators were Soochow University's graduate students, who had all been trained beforehand to be fully familiar with the test items and procedures. Classroom physical education teachers were present to assist. Before the start of the test, the administrators demonstrated the sequence and combination of the test items in their entirety and allowed each participant to practice once. During the actual testing, one administrator used a stopwatch to record the time, while another assisted with the completion of the tests and provided safety protection.

The TCFMST includes three dimensions: locomotive skills, body control skills, and manipulative skills, with a total of 10 items: Single and double feet alternating jumping grid, jumping through hoops on one leg, crawling with hands and feet, side slip step, sideward roll, walking forward on the balance beam, turning around and jumping, throwing sandbags, spot kick, and catching sandbags with both hands. The test methods and precautions are shown in Supplementary Table S11.

The testing period was from 20 November 2023 to 15 December 2023, excluding Saturdays and Sundays. To avoid the practice effect, retesting was conducted on a random selection of 80 children from the same group, 2 weeks after the completion of the formal testing (40) to analyze the test–retest reliability of the TCFMST. The retest period was from 4 January 2024 to 5 January 2024.

#### TGMD-3

The Test of Gross Motor Development, Third Edition (TGMD-3) is the latest version of the TGMD series and is widely used internationally with high reliability and validity (41–43). Therefore, TGMD-3 was chosen as a tool to validate the concurrent validity of the TCFMST. The TGMD-3 includes specific test items that assess locomotor skills such as “running,” “galloping,” “hopping on one foot,” “jump running,” “standing long jump,” and “sidestepping.” It also evaluates ball skills through items such as “striking a stationary ball with both hands,” “volleying a falling ball with the forehand,” “catching a ball with both hands,” “kicking a ball,” “bouncing a ball on one hand in place,” “overhand throwing,” and “underhand tossing.” Additionally, recordings are made of the students' movements, and testers score the students' actions based on these video assessments. The test administrators were trained on TGMD-3 prior to the study. By using both TCFMST and TGMD-3 to test the same group of subjects simultaneously, the concurrent validity of the TCFMST was examined. Eighty students aged 7 to 8 years were selected from two primary schools in Suzhou city, with an equal proportion of boys and girls and a similar age distribution. The concurrent administration of two different tests for the development of fundamental motor skills on the 80 subjects allowed for the correlation analysis of the two sets of test data.

### Data analysis

During the development process of TCFMST, relevant data were primarily input and analyzed using software such as Excel and SPSS 26.0. The application of specific methods is evident in several aspects: (1) During the revision and improvement phase of the test, data from Delphi expert consultation forms are processed and analyzed using measures such as mean, SD, coefficient of variation, Kendall's coordination coefficient, Pearson's correlation analysis, and exploratory factor analysis to test the structural validity of the pilot test. (2) During the validation phase of the test, Pearson's correlation analysis is used to examine the test's test–retest reliability and concurrent validity.

## Results

### Dependability of expert consultation

#### The authority of the expert

The authority of experts greatly influences the reliability of consultations, which is primarily determined by their judgmental basis and familiarity with the subject matter. Among these, the coefficient of influence of judgmental basis is denoted as Ca, the coefficient of familiarity with the question is denoted as Cs, and the authority coefficient of the expert, Cr, is calculated as the average of Ca and Cs, with Cr = (Ca + Cs)/2 (44). The specific quantification of judgmental basis and familiarity with the subject matter can be found in Supplementary Tables S2, S3, respectively.

When Ca = 1, it indicates that the judgmental basis has a significant impact on the expert; when Ca = 0.8, it suggests a moderate impact; and when Ca = 0.6, the impact is considered small. Typically, a Cr > 0.7 is regarded as indicating a higher reliability of the expert consultation. In the first round, with Ca = 0.954, Cs = 0.877, and Cr = 0.916, it is evident that the reliability of the first round of expert consultations is relatively high.

#### Degree of coordination among experts

The degree of experts' consensus is used to reflect whether there is significant disagreement among experts on various test items, as well as whether there are conflicting opinions (45). Typically, this degree is represented by Kendall's W, a coefficient that measures the consistency of m experts' evaluations on n items. The coefficient's value ranges from 0 to 1, with higher values indicating a higher degree of consensus (44). Additionally, chi-square tests are used to further assess the degree of consensus, with the significance level α set at 0.05.

The first round of expert consensus coefficient was 0.280, indicating a low degree of consensus and a significant divergence in expert opinions. In contrast, the second round's consensus coefficient was 0.565, signifying an improvement in coordination compared to the first round. This suggests that the expert opinions were converging toward a consensus, and it met the standard for terminating expert consultation (W > 0.500), indicating that the second round of expert results was acceptable and that the expert consultation could be concluded. Moreover, Kendall's W-test for consensus among the two rounds of experts was statistically significant (*p* < 0.05) (Supplementary Table S4).

### Results of the delphi expert consultation

#### Principles of project selection

Experts scored the recommendation level of each test item, with the “recommendation level” assigned a range of “1 to 5 points.” Higher scores indicate a higher level of recommendation. The recommendation level considers several aspects: (1) measuring the importance and suitability of each item within the TCFMST; (2) the operational feasibility of each item in the Chinese school physical education environment (mainly considering factors such as the availability of facilities, equipment, and time); and (3) the reasonableness of using timing for the evaluation of the items. Based on the expert scores, the arithmetic mean, SD, and coefficient of variation were calculated for each category of test items.

The selection criteria for the test items are as follows: (1) If the arithmetic mean is ≥3.5 and the coefficient of variation is ≤ 20%, the item is retained; (2) If the arithmetic mean is ≤ 3.5 and the coefficient of variation is ≥20%, the test item is deleted; (3) If the arithmetic mean is ≤ 3.5 and the coefficient of variation is ≤ 20%, or if the arithmetic mean is ≥3.5 and the coefficient of variation is ≥20%, the item requires further discussion by the research team. If consensus is still not reached, expert opinions are sought. Furthermore, the research team also modifies the items based on specific suggestions or recommendations made by the experts. Additionally, any different opinions are reported in the expert consultation form, along with explanations.

#### Expert consultation improvement results

The results of the first and second rounds of expert consultation are presented in Supplementary Tables S5, S6, respectively, and the final determined items after expert consultation improvement are shown in Table 2.

**Table 2:** Ultimate outcome of expert consultation for improvement.

**Dimension**	**Test items**
Body control skills	Step by step walking
	Walking forward on the balance beam
	Sideward roll
	Turn around and jump
	Throwing sandbags
	Throw and catch the ball with both hands on the spot
Manipulative skills	Bounce the ball with one hand on the spot
	Spot kick
	Catch sandbags with both hands
	Side slip step
	Jump through hoops on one leg
Locomotive skills	Continuous hoop jumping with both feet
	Single and double feet alternating jumping grid
	Crawling with hands and feet

#### The distinguishability of the test

The item-total correlation method is a commonly used approach to assess the discriminate power of test items. It does so by calculating the correlation coefficient between the score of each item and the total score of the scale to evaluate the discriminate power. A high correlation between the item score and the total score indicates that the item can effectively differentiate the overall level of the subjects. Test items with high discriminate power can distinguish subjects with different levels of motor development. A discriminate power value of 0.4 or above is generally considered very good, 0.30 to 0.29 is good, 0.20 to 0.29 is acceptable, and below 0.20 is poor. The discriminate power of the TCFMST test items is as follows (Table 3).

**Table 3:** Discrimination of each test item.

	**Body control skills total score**	**Manipulative skills total score**	**Locomotive skills total score**	**Test total score**
Step by step walking	0.885^*^			0.821^*^
Walking forward on the balance beam	0.713^*^			0.653^*^
Sideward roll	0.627^*^			0.623^*^
Turn around and jump	0.527^*^			0.534^*^
Throwing sandbags		0.899^*^		0.547^*^
Bounce the ball with one hand on the spot		0.171		0.177
Spot kick		0.556^*^		0.355^*^
Catch sandbags with both hands		0.719^*^		0.611^*^
Side slip step			0.621^*^	0.301^*^
Jump through hoops on one leg			0.756^*^	0.332^*^
Continuous hoop jumping with both feet			0.056	0.054
Single and double feet alternating jumping grid			0.847^*^	0.386^*^
Crawling with hands and feet			0.704^*^	0.392^*^

* Indicates *p* < 0.05.

#### Standardization test

Test standardization refers to the consistency in the development, administration, scoring, and interpretation of test results. For the TCFMST, standardization ensures that the results obtained from the fundamental motor skills test can be compared without being influenced by external conditions such as the test site, equipment, implementation process, and scoring standards. The differences in test scores among subjects are attributed to the individual differences among the subjects themselves.

The standardization of the TCFMST is primarily manifested in the following four aspects: (1) standardization of the test procedure. In terms of the specific sequence and combination of test items, the principles of safety, scientificity, and fluidity are adhered to, while also considering the overall utilization of the test site, ensuring a smooth and natural transition in the overall process; (2) standardization of test site and equipment. The test methods, site layout, and equipment specifications are the most crucial aspects of a fundamental motor skills development test. Variations in these factors can significantly impact the test results. The equipment required for the TCFMST is standard and commonly found in primary and secondary schools, including stopwatches, footballs, and basketballs. Moreover, standardized site layout diagrams have been developed to enhance test efficiency and ensure the feasibility of the test; (3) The standardization of the test explanation and demonstration is mainly reflected in four aspects, including the explanation and demonstration before the test, the verbal prompts during the test, the record of the results after the test, and the test scenario. For example, in the demonstration of “walking forward on the balance beam,” the testers need to show the subjects that their bodies are facing the direction of the finish line during the test, their hands are held sideways, and they are actively moving toward the other end of the balance beam, etc. They also need to remind the subjects that if they make a mistake in the middle of the test, they need to start the test again. At the same time, the testers need to demonstrate the action. (4) Standardize the verbal prompts of the test. The testers can give verbal prompts to the subjects who are about to be tested or are being tested, but the content of the verbal prompts is only limited to the name of the test action, the method of the test, and the attention to safety matters. For example, the testers can give verbal prompts to the subjects who are throwing sandbags, with the verbal expression “the next item, throwing sandbags” “it's a miss., and then again!” “test passed,” “be safe,” and so on.

### Significance test of difference

An independent samples *t*-test was conducted to analyze the differences in FMS test scores between genders (Table 4).

**Table 4:** Gender differences in FMS development analysis.

**Age**	**Gender**	**Sample**	***M ±SD***	***t-* Value**	***p-* Value**
7	Male	243	58.44 ± 13.221	−0.998	0.320
	Female	247	60.21 ± 11.151		
8	Male	261	50.57 ± 10.249	−4.263	0.000
	Female	254	56.18 ± 8.626		

Subsequently, with the FMS test scores of students as the dependent variable and age as the independent variable, a one-way analysis of variance (ANOVA) was conducted to analyze the differences in the development of fundamental motor skills among students of different grades (Table 5).

**Table 5:** Age differences in FMS development analysis.

**Age**	**Sample**	***M ±SD***	***F*-Value**	***p*-Value**
7	490	59.33 ± 12.289	28.822	<0.001
8	515	53.34 ± 9.874		
Total	1,005	56.26 ± 11.481	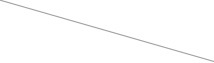	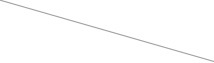

### Test–retest reliability

Two weeks after the completion of the TCFMST test, 80 subjects were randomly selected from the test group for retesting, and the results were subjected to Pearson's correlation analysis (Table 6).

**Table 6:** Test–retest reliability coefficients.

**Dimension**	**Test items (*n* = 80)**	**The first test**	**The second test**	**Correlation coefficient**	***p-*Value**
		**M** ±**SD**	**M** ±**SD**		
Body Control Skills	Step by step walking	19.78 ± 4.478	18.88 ± 3.797	0.801	<0.001
	Walking forward on the balance beam	5.63 ± 1.844	5.79 ± 1.412	0.871	
	Sideward roll	4.25 ± 0.968	4.22 ± 0.676	0.908	
	Turn around and jump	2.31 ± 0.558	2.26 ± 0.458	0.943	
Manipulative Skills	Throwing sandbags	6.19 ± 2.156	6.47 ± 1.938	0.925	<0.001
	Bounce the ball with one hand on the spot	2.90 ± 0.153	3.06 ± 0.132	0.872	
	Spot kick	3.16 ± 1.311	3.05 ± 0.766	0.899	
	Catch sandbags with both hands	1.75 ± 0.456	1.98 ± 0.248	0.851	
Locomotive Skills	Side slip step	4.93 ± 0.432	4.63 ± 0.374	0.799	<0.001
	Jump through hoops on one leg	3.37 ± 1.035	3.33 ± 0.637	0.931	
	Continuous hoop jumping with both feet	4.16 ± 0.893	4.16 ± 0.557	0.881	
	Single and double feet alternating jumping grid	8.80 ± 0.803	7.97 ± 0.659	0.905	
Test total score		71.08 ± 9.186	69.60 ± 6.846	0.926	<0.001

The retest reliability of the test items in the TCFMST is high. Additionally, the correlation coefficients between the total scores of the three dimensions and the total test scores in the first and second tests are 0.862, 0.937, 0.839, and 0.926, respectively. This indicates that the results of the test items are highly stable.

### Validity

#### Construct validity

Exploratory factor analysis was conducted to determine whether the structure of the TCFMST aligns with the conceptual model of motor development theory. The analysis aimed to confirm if the inherent components of the test match the anticipated dimensions. The specific criteria for judgment are as follows: (1) The common factors should correspond to the hypothesized composition domains, and their cumulative variance contribution rate should be at least 40%; (2) Each item should have a high loading value on one of the common factors, i.e., >0.4; and (3) The common factor variance should all be >0.4.

First, the Kaiser–Meyer–Olkin (KMO) and Bartlett's test of sphericity were applied to determine if the items of the TCFMST were suitable for factor analysis. Bartlett's test of sphericity is used to judge whether the correlation matrix is an identity matrix. The KMO test assesses the partial correlation among variables, with values ranging from 0 to 1. A value closer to 1 indicates stronger partial correlations and suggests that the structure is more suitable for factor analysis. Generally, a KMO value below 0.5 indicates that the structure is not suitable for factor analysis (Supplementary Table S7). This rejects the assumption of independence among variables. Therefore, the items are suitable for factor analysis.

Through principal component analysis, following Kaiser's criterion, the number of factors to be extracted is determined based on the criterion that the factor eigenvalue must be >1.0. After rotation using the maximum variance method, three factors were obtained (Supplementary Table S8). The eigenvalues of the factors are 3.422, 2.004, and 1.095, respectively, which explain 31.11%, 18.22%, and 9.95% of the total variance, with a cumulative variance contribution rate of 59.28%. According to the rotated component matrix (Supplementary Table S9), the factor “Step by step walking” spans two components, and its factor loadings are <0.5. Therefore, it was deleted.

The ensuing factor analysis is conducted with a consistent methodological approach as utilized earlier in the study (Supplementary Table S10).

**Figure 2:**
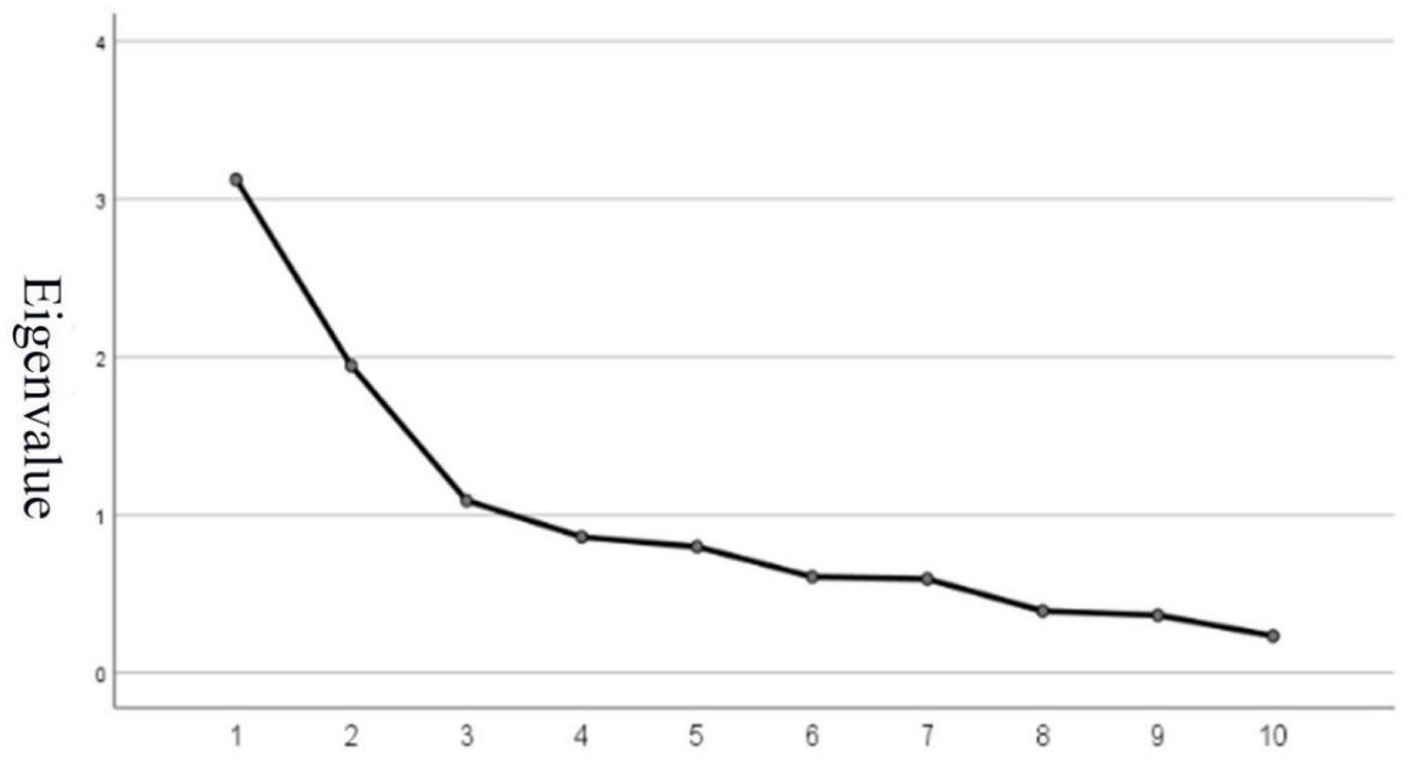
Gravel inspection curve.

The results, as shown in Supplementary Table S10, indicate that the KMO value is 0.675, which is >0.5. The significance value of Bartlett's test of sphericity is 0.000, which is <0.05. This indicates that the adjusted TCFMST items are suitable for factor analysis. Through principal component analysis with maximum variance rotation, three factors were obtained. Additionally, as indicated by the scree plot (Figure 2), the curve began to flatten after the third factor, suggesting that extracting three factors is appropriate. This aligns with the preliminary proposed structure of three dimensions for fundamental motor skills from the earlier stages. As shown in Table 7, the eigenvalues are 23.12%, 22.89%, and 15.57%, respectively, with a cumulative variance contribution rate of 61.58%, which is higher than the previous round.

**Table 7:** Characteristics of each factor and cumulative variance contribution rate.

	**Initial feature value**			**Sum of squares of revolving loads**		
**Common divisor**	**Characteristic value**	**Variance contribution rate (%)**	**Cumulative variance contribution rate (%)**	**Characteristic value**	**Variance contribution rate (%)**	**Cumulative variance contribution rate (%)**
1	3.125	31.248	31.248	2.312	23.121	23.121
2	1.943	19.431	50.679	2.289	22.888	46.009
3	1.090	10.903	61.582	1.557	15.573	61.582

According to the factor loadings obtained from the rotated component matrix, all factor loadings are >0.5 (Table 8). Considering the characteristics of items included in each common factor, the first common factor exhibits significant loadings on “walking forward on a balance beam,” “sideward roll,” and “turn around and jump,” thus being identified as body control skills. The second common factor shows substantial loadings on “throwing sandbags,” “Spot kick,” and “catching beanbags with both hands,” indicating manipulative skills. The third common factor demonstrates considerable loadings on “Single and double feet alternating jumping grid,” “jump through hoops on one leg,” “crawling with hands and feet,” and “Side slip step,” suggesting locomotive skills.

**Table 8:** Factor loading matrix after rotation.

**Items**	**Common factor**		
	**1**	**2**	**3**
Single and double feet alternating jumping grid	0.800	0.232	0.013
Jump through hoops on one leg	0.733	0.093	0.013
Crawling with hands and feet	0.695	0.153	−0.089
Side slip step	0.688	−0.027	0.187
Sideward roll	0.116	0.855	0.109
Walking forward on the balance beam	0.068	0.793	0.066
Turn around and jump	0.250	0.680	0.116
Throwing sandbags	−0.248	0.388	0.715
Spot kick	0.173	−0.131	0.711
Catch sandbags with both hands	0.080	0.460	0.684

**Figure 3:**
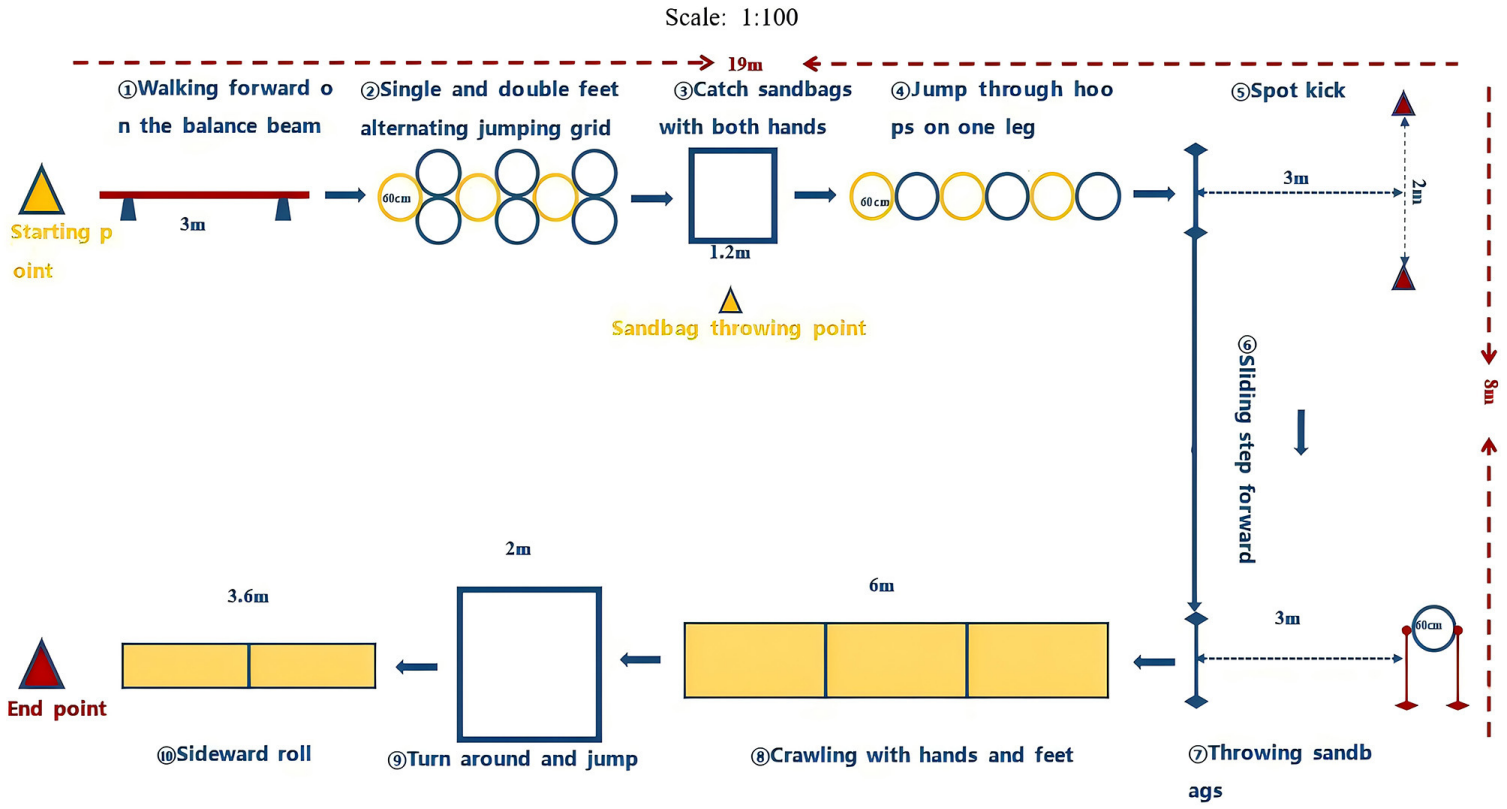
Schematic diagram of the TCFMST site.

At this point, the relatively well-established TCFMST project has been developed, along with the planning of TCFMST's testing sites (Figure 3) and the standardization of the testing process (Supplementary Table S11).

#### Concurrent validity

The TCFMST and TGMD-3 were used simultaneously to test the fundamental motor skill development of the same group of subjects. The scores from both tests were subjected to correlation analysis to obtain the concurrent validity coefficient of the TCFMST, thereby testing its accuracy in reflecting the development of the subjects' fundamental motor skills (Table 9).

**Table 9:** Correlation between TCFMST and TGMD-3 scores.

**Variable**	**TCFMST results**		
	**Total (*****n** =* **80)**	**Male (*****n** =* **40)**	**Female (*****n** =* **40)**
TGMD-3 total score	−0.510^*^	−0.521^*^	−0.466^*^

* Indicates *p* < 0.05.

The results show that there is a moderate correlation between the TCFMST test results and the total score of TGMD-3. Additionally, there is also a moderate correlation between the TCFMST results of different genders and the total score of TGMD-3. This indicates that the concurrent validity of the TCFMST is high and it possesses high accuracy.

## Discussion

### Differences in children's FMS levels among different genders or ages

Our study found that boys demonstrate better development in fundamental motor skills compared to girls (Table 4). This finding is consistent with other research results: boys scored higher in FMS and physical functioning than girls, and girls scored higher in physical health, emotional, and school functioning than boys (46). For 7-year-olds, there was no significant difference in the level of basic motor skill development between boys and girls (*p* > 0.05). In contrast, for 8-year-olds, a very significant difference was observed in the development of basic motor skills between the genders (*p* < 0.01). Children mature at different rates, and these rates can affect motor skill development. At age 7, boys and girls might be at similar stages of physical and neurological development, leading to comparable FMS scores. By age 8, however, boys might be experiencing a growth spurt or faster neuromuscular development, which could result in better motor performance (47).

There is a significant difference in the level of fundamental motor skill development among students of different grades, with 7-year-old students exhibiting lower levels of development compared to their 8-year-old counterparts (Table 5). The developmental sequence model of motor skill proficiency posits that children's FMSs gradually improve with age, a concept consistent with our study's findings. Our research once again confirms the core tenet of the developmental sequence model, which is that children's FMS progressively advance as they grow older (48).

### Comparison of the TCFMST with other FMS assessments

#### Purpose and applicable objects of the test

The original purpose of FMS assessments was to screen and diagnose motor abilities in special groups of children, such as those with typical developmental patterns, including children with brain injuries, hearing impairments, and heart conditions (19); or as in the case of the Bruininks–Oseretsky test (BOT) of motor proficiency, which is primarily used to assess whether children have motor deficits or disabilities (20). Currently, FMS assessments not only focus on screening for motor issues in special children but also emphasize promoting the development of motor skills in typically developing children. For example, the Canadian Assessment of Physical Literacy (CAPL) includes the Children's Assessment of Movement Skill Abilities (CAMSAs), which aims to evaluate the movement skills of 8- to 12-year-old children and their ability to adapt to environmental changes by combining simple movement skills with executing more complex ones (27).

The TCFMST differs from existing assessments in terms of the purpose and scope of applicable subjects. In terms of application purpose, the TCFMST aims to screen for movement risks and monitor motor development within the school physical education environment. In terms of the scope of applicable subjects, the test narrows the age range, focusing on the development of FMSs in children aged 7 to 8 years.

#### Testing site and equipment

The TCFMST requires an 18-m-long by 9-m-wide area for arranging and combining various test items to form a test track. It is worth noting that most existing indoor and outdoor tracks and courts in schools can be used as test sites, and there are no special requirements for the site, making it highly practical. In terms of the specifications of the testing equipment, items such as balance beams, sponge mats, agility hoops, soccer balls, basketballs, marker cones, and marker poles are commonly used in school physical education. The inclusion of beanbags, a traditional Chinese folk sports equipment, adds both fun and local characteristics to the test.

Other existing assessments typically require specific venues and custom-made equipment. For example, the “Backward Balance Walking” test in KTK requires one beam for each side, each measuring 3 meters in length, 3 cm in height, and 6 cm, 4.5 cm, and 3 cm in width, respectively (22). The “One-Leg Jump” test requires 12 foam boards, each measuring 50 cm in length, 20 cm in width, and 5 cm in height. In TGMD-3, the “Both-Handed Strike of a Stationary Ball” requires a special ball, a bat, and a stand, while the “Overhand Strike of a Thrown Ball” requires a special ball and a racket (30).

In contrast, the TCFMST can fully utilize the existing venues and equipment in schools. The choice of the testing site is flexible and convenient, effectively reducing conflicts with teaching areas. The test equipment can be selected from what is available on site, thus enhancing test efficiency.

#### Testing method and test duration

Existing FMS assessments include three types of evaluation methods: outcome-oriented, process-oriented, and a combination of both. The TCFMST uses a “track-style” outcome-oriented evaluation, where test items are arranged and combined in a test track. The subject is required to complete the track as quickly as possible, with completion time being the sole measurement parameter. In terms of test duration, subjects typically take only 1 min on average to complete the “track-style” test. In contrast, other outcome-oriented assessments usually require longer time, such as the BOT-2 test, which takes 45–60 min (19), and assessments such as Movement-ABC-2 and KTK typically take approximately 20 min (23, 24).

It should be noted that there is a moderate correlation between the TCFMST and TGMD-3, indicating that although the test takes less time, it has high concurrent validity and accuracy.

### Advantages and limitations of the TCFMST

The TCFMST demonstrates significant advantages, mainly in terms of efficiency, ease of operation, interest, specificity, and economic practicality.

First, in terms of efficiency, the TCFMST is characterized by its short test duration, with an average of only 1 min per person. This high efficiency is particularly advantageous in large-scale testing environments, allowing for the rapid and effective assessment of a large number of students within a limited time. Compared to other assessments (49), this improvement in time efficiency is a significant advancement, enhancing the speed of assessment while minimizing the extensive use of teaching time.

Second, the ease of operation is another highlight of the TCFMST. It adopts an “outcome-oriented” evaluation method, with test duration being the sole criterion for measuring the subject's level, thus avoiding discrepancies in scoring results due to the personal experience and subjective judgment of the tester. Additionally, the design of the test avoids complex setups and the need for special equipment, ensuring that the test items are easy to understand and execute, reducing the difficulty for teachers in conducting tests and minimizing the possibility of execution errors, thus making the test results more reliable and accurate.

Moreover, the design of interest in the TCFMST is crucial for increasing students' engagement and enthusiasm. Incorporating traditional Chinese physical activity games such as “throwing sandbags” and “hopscotch” into the test not only stimulates students' interest but also reduces the psychological pressure associated with the “exam” nature of the test, resulting in a more authentic display of their motor abilities. This design philosophy aligns with modern educational requirements, which aim to enhance educational effectiveness by stimulating students' interest in learning.

Finally, the economic practicality is another advantage of the “track-style” test. The equipment required for the test is common or provided in primary and secondary schools, eliminating the need for additional investment, which greatly reduces the economic threshold for testing. This design makes the test not only efficient and accurate but also economical and practical, making it suitable for widespread promotion and application.

In summary, the TCFMST demonstrates significant advantages in various aspects, not only enhancing the efficiency of assessment, reducing operational difficulty, and increasing student engagement but also closely integrating with course content while maintaining economic practicality. These advantages make it an effective tool for assessing students' motor skills and deserve wide application in educational practice.

This study selected students from urban primary schools for the validation phase, and future research could further expand the sample to include students from other regions and rural areas. Additionally, the target group for this test is 7- to 8-year-old children, and future research plans to extend its application to other age groups to monitor the proficiency of FMS. However, this would require further reliability and validity testing.

In general, the TCFMST has significant advantages in terms of efficiency and practicality, but it also has limitations in terms of the scope of testing and the depth of feedback. In practical application, it is necessary to consider these factors comprehensively to fully exploit the advantages of the test while trying to compensate for its limitations.

## Conclusion

FMS is a fundamental element of the overall development of children. Accurate assessment and timely monitoring of children's FMS development are crucial for enhancing their health outcomes. The development and validation of the TCFMST offer a refined tool for assessing motor skills in children, tailored to the Chinese educational context. Our study confirms the TCFMST's strong psychometric properties, with robust reliability and validity, providing a nuanced evaluation of children's fundamental motor skills. The study selected items from existing FMS assessments, textbooks, physical education and health standards, and children's movement guidelines to construct a pool of test items. Subsequently, the items were screened and optimized using the Delphi method. Finally, the feasibility, discrimination, difficulty, reliability, and validity of the constructed test were examined using testing methods. The TCFMST has good difficulty, discrimination, reliability, and validity, while also featuring strong operability, short duration, and high interest. It can effectively assess students' fundamental motor skill development levels and can serve as an important tool for monitoring fundamental motor skill levels in school education contexts. The TCFMST's potential for adaptation to different educational contexts and its sensitivity to developmental changes position it as a valuable asset in physical education.

However, the scope of the test and the depth of feedback it provides are areas that require further refinement. The test's focus on children aged 7 to 8 presents an opportunity for future research to extend its application to other age groups, thereby broadening its utility and allowing for more comprehensive monitoring of the development of fundamental motor skills.

## Data Availability

The original contributions presented in the study are included in the article/Supplementary material, further inquiries can be directed to the corresponding author.
