# Supplementary material for: “Track style” children's fundamental movement skills test: construction and verification of an efficient evaluation system
Source: Front Public Health. 2024 Aug 19;12:1437473. doi: 10.3389/fpubh.2024.1437473 (PMC11369310; doi:10.3389/fpubh.2024.1437473)
Supplement: Supplementary file 1 [file Table_1.DOCX]

| **Supplementary Material 1**  Table S1 Basic composition of advisory experts | | | |
| --- | --- | --- | --- |
| Item | Basic information | Number (persons) | Composition ratio（%） |
| Sex | Male | 9 | 64.29 |
|  | Female | 5 | 35.71 |
| Age | 30～40 | 5 | 35.71 |
|  | 41～50 | 4 | 28.57 |
|  | >50 | 5 | 35.71 |
| Title | Intermediate | 2 | 14.29 |
|  | Associate professor | 6 | 42.86 |
|  | Professor | 6 | 42.86 |
| Education attainment | Undergraduate | 1 | 7.14 |
|  | Master | 3 | 21.43 |
|  | Doctor | 10 | 71.43 |

| **Supplementary Material 2**  Table S2 Criteria for Expert Judgment and Their Quantified Values | | | |
| --- | --- | --- | --- |
| Basis for judgment | The degree of influence on expert judgment | | |
|  | Big | Middle | Small |
| Theoretical analysis | 0.3 | 0.2 | 0.1 |
| Practical experience | 0.5 | 0.4 | 0.3 |
| Understanding from peers both domestically and internationally | 0.1 | 0.1 | 0.1 |
| Intuition | 0.1 | 0.1 | 0.1 |

| **Supplementary Material 3**  Table S3 Quantified Values of Experts' Familiarity with the Issue | |
| --- | --- |
| Familiarity | Cs |
| Very familiar | 1 |
| Quite Familiar | 0.8 |
| Familiar | 0.6 |
| Not very familiar | 0.4 |
| Unfamiliar | 0.2 |

| **Supplementary Material 4**  Table S4 Coefficient of Expert Consultation Opinion Coordination | | | |
| --- | --- | --- | --- |
| Number of rounds | Coordinating coefficient | Chi-square | P value |
| First round | 0.280 | 145.424 | 0.000 |
| Second round | 0.565 | 142.335 | 0.000 |

| **Supplementary Material 5**  Table S5 Results of the First Round of Expert Consultation | | | | | | | | | | | | | |
| --- | --- | --- | --- | --- | --- | --- | --- | --- | --- | --- | --- | --- | --- |
| Dimension | | | Serial number | | Test items | | Average value（s） | Standard deviation（s） | | Coefficient of Variation | | Modification | |
| (A) Body Control Skills | | | A1 | | Tall and Dwarf People | | 3.462 | 1.050 | | 0.30 | | Delete | |
|  |  |  | A2 | | Tiptoe walking | | 3.077 | 0.954 | | 0.31 | | Delete | |
|  |  |  | A3 | | Step by step walking | | 4.154 | 0.555 | | 0.13 | | Be selected | |
|  |  |  | A4 | | forward roll | | 3.615 | 1.044 | | 0.29 | | Delete | |
|  |  |  | A5 | | Walking forward on the balance beam | | 4.692 | 0.480 | | 0.10 | | Be selected | |
|  |  |  | A6 | | Walking sideways on the balance beam | | 3.154 | 0.899 | | 0.28 | | Delete | |
|  |  |  | A7 | | Going straight backwards | | 3.769 | 0.832 | | 0.22 | | Continue to inquire | |
|  |  |  | A8 | | In situ different side hand and foot clap | | 3.385 | 1.044 | | 0.31 | | Delete | |
|  |  |  | A9 | | Sideward roll | | 3.539 | 0.776 | | 0.22 | | Continue to inquire | |
|  |  |  | A10 | | Turn around and jump | | 3.846 | 0.801 | | 0.21 | | Continue to inquire | |
| (B) Manipulative Skills | | | B1 | | Throwing sandbags | | 4.308 | 0.751 | | 0.17 | | Be selected | |
|  |  |  | B2 | | Underhand roll | | 4.000 | 1.000 | | 0.25 | | Continue to inquire | |
|  |  |  | B3 | | Throw and catch the ball with both hands on the spot | | 3.539 | 0.967 | | 0.27 | | Continue to inquire | |
|  |  |  | B4 | | Throw and catch the ball with one hand on the spot | | 3.923 | 0.494 | | 0.13 | | Be selected | |
|  |  |  | B5 | | Move a ball or other object | | 3.308 | 1.032 | | 0.31 | | Delete | |
|  |  |  | B6 | | Bounce the ball with one hand on the spot | | 4.077 | 0.760 | | 0.19 | | Be selected | |
|  |  |  | B7 | | Dribble | | 3.923 | 1.115 | | 0.28 | | Delete | |
|  |  |  | B8 | | Badminton Racket Bounce on the spot | | 3.308 | 0.855 | | 0.26 | | Delete | |
|  |  |  | B9 | | Strike rebound ball | | 3.000 | 0.913 | | 0.30 | | Delete | |
|  |  |  | B10 | | Kick the ball against the wall | | 3.462 | 0.776 | | 0.22 | | Delete | |
|  |  |  | B11 | | Spot kick | | 3.769 | 0.927 | | 0.25 | | Be selected | |
| (C) Locomotive Skills | | | C1 | | Sliding step forward | | 3.692 | 1.032 | | 0.28 | | Delete | |
|  |  |  | C2 | | Sideslip step | | 3.846 | 0.899 | | 0.23 | | Continue to inquire | |
|  |  |  | C3 | | S-Running | | 3.462 | 0.967 | | 0.28 | | Delete | |
|  |  |  | C4 | | Z-Running | | 3.846 | 0.689 | | 0.18 | | Be selected | |
|  |  |  | C5 | | Sprint | | 3.077 | 0.954 | | 0.31 | | Delete | |
|  |  |  | C6 | | 10 Meters turnaround run | | 3.462 | 1.050 | | 0.30 | | Delete | |
|  |  |  | C7 | | Continuous running across obstacles | | 3.154 | 0.801 | | 0.25 | | Delete | |
|  |  |  | C8 | | Jump through hoops on one leg | | 4.462 | 0.519 | | 0.12 | | Be selected | |
|  |  |  | C9 | | Continuous hoop jumping with both feet | | 3.923 | 0.641 | | 0.16 | | Be selected | |
|  |  |  | C10 | | Continuous tiptoe jumping | | 3.385 | 0.870 | | 0.26 | | Delete | |
|  |  |  | C11 | | Continuous pad jump | | 3.615 | 1.044 | | 0.29 | | Delete | |
|  |  |  | C12 | | Continuous jumping jacks | | 4.000 | 1.000 | | 0.25 | | Continue to inquire | |
|  |  |  | C13 | | Single and double feet alternating jumping grid | | 4.385 | 0.768 | | 0.18 | | Continue to inquire | |
|  |  |  | C14 | | Continuous left and right side jumps on both feet | | 3.462 | 0.877 | | 0.25 | | Delete | |
|  |  |  | C15 | | Jump rope with both feet | | 3.077 | 0.954 | | 0.31 | | Delete | |
|  |  |  | C16 | | Jump the soft square bag with both feet continuously | | 4.000 | 0.577 | | 0.14 | | Be selected | |
|  |  |  | C17 | | Jump the soft square bag with one foot continuously | | 4.077 | 0.954 | | 0.23 | | Continue to inquire | |
|  |  |  | C18 | | Crawling with hands and feet | | 4.308 | 0.630 | | 0.15 | | Be selected | |
|  |  |  | C19 | | Crawling with hands and knee | | 3.000 | 0.913 | | 0.30 | | Delete | |
|  |  |  | C20 | | Crawl | | 4.077 | 0.862 | | 0.21 | | Continue to inquire | |
| **Supplementary Material 6**  Table S6 Results of the Second Round of Expert Consultation | | | | | | | | | | | | |  |
| Dimension | Serial number | | Test items | | Average value（s） | | | Standard deviation（s） | | Coefficient of Variation | | Modification |  |
| (A) Body Control Skills | A3 | | Step by step walking | | 3.833 | | | 0.389 | | 0.102 | | Be selected |  |
|  | A5 | | Walking forward on the balance beam | | 4.500 | | | 0.522 | | 0.116 | | Be selected |  |
|  | A7 | | Going straight backwards | | 3.417 | | | 0.900 | | 0.264 | | Delete |  |
|  | A9 | | Sideward roll | | 4.667 | | | 0.492 | | 0.106 | | Be selected |  |
|  | A10 | | Turn around and jump | | 3.583 | | | 0.515 | | 0.144 | | Be selected |  |
| (B) Manipulative Skills | B1 | | Throwing sandbags | | 3.917 | | | 0.515 | | 0.131 | | Be selected |  |
|  | B2 | | Underhand roll | | 3.333 | | | 0.778 | | 0.234 | | Delete |  |
|  | B3 | | Throw and catch the ball with both hands on the spot | | 4.583 | | | 0.515 | | 0.112 | | Be selected |  |
|  | B4 | | Throw and catch the ball with one hand on the spot | | 2.833 | | | 0.389 | | 0.137 | | Delete |  |
|  | B6 | | Bounce the ball with one hand on the spot | | 4.167 | | | 0.389 | | 0.093 | | Be selected |  |
|  | B11 | | Spot kick | | 4.250 | | | 0.452 | | 0.106 | | Be selected |  |
| (C) Locomotive Skills | C2 | | Sideslip step | | 4.333 | | | 0.492 | | 0.114 | | Be selected |  |
|  | C4 | | Z-Running | | 3.417 | | | 0.669 | | 0.196 | | Delete |  |
|  | C8 | | Jump through hoops on one leg | | 4.500 | | | 0.522 | | 0.116 | | Be selected |  |
|  | C9 | | Continuous hoop jumping with both feet | | 3.750 | | | 0.452 | | 0.121 | | Be selected |  |
|  | C12 | | Continuous jumping jacks | | 3.417 | | | 0.793 | | 0.232 | | Delete |  |
|  | C13 | | Single and double feet alternating jumping grid | | 4.417 | | | 0.669 | | 0.151 | | Be selected |  |
|  | C16 | | Jump the soft square bag with both feet continuously | | 2.917 | | | 0.289 | | 0.099 | | Delete |  |
|  | C17 | | Jump the soft square bag with one foot continuously | | 4.250 | | | 0.452 | | 0.106 | | Delete |  |
|  | C18 | | Crawling with hands and feet | | 4.083 | | | 0.515 | | 0.126 | | Be selected |  |
|  | C20 | | Crawl | | 3.333 | | | 0.778 | | 0.234 | | Delete |  |

| **Supplementary Material 7**  Table S7 KMO Value and Bartlett's Sphericity Test Results | | | | | | | | | | | | | | | |  |
| --- | --- | --- | --- | --- | --- | --- | --- | --- | --- | --- | --- | --- | --- | --- | --- | --- |
| Inspection Items | | | | Inspection indicators | | | | | | Statistical Value | | | | | |  |
| KMO | | | |  | | | | | | 0.698 | | | | | |  |
| Bartlett’ sphericity test | | | | Chi-square value | | | | | | 335.729 | | | | | |  |
|  | | | | Degree of freedom, | | | | | | 55 | | | | | |  |
|  | | | | Statistical significance | | | | | | 0.000 | | | | | |  |
| **Supplementary Material 8**  Table S8 Characteristics of Each Factor and Cumulative Variance Contribution Rate | | | | | | | | | | | | | | | | |
|  | Initial Feature Value | | | | | | | Sum of Squares of Revolving Loads | | | | | | | | |
| Common divisor | Characteristic value | Variance Contribution Rate（%） | | | Cumulative variance contribution rate（%） | | | Characteristic value | | | Variance contribution rate（%） | | | Cumulative variance contribution rate（%） | | |
| 1 | 3.422 | 31.112 | | | 31.112 | | | 2.434 | | | 22.127 | | | 22.127 | | |
| 2 | 2.004 | 18.220 | | | 49.332 | | | 2.321 | | | 21.097 | | | 43.223 | | |
| 3 | 1.095 | 9.951 | | | 59.283 | | | 1.767 | | | 16.060 | | | 59.283 | | |
| **Supplementary Material 9**  Table S9 Factor Loading Matrix after Rotation | | | | | | | | | | | | | | |  |  |
| Items | | | | | | Common Factor | | | | | | | | |  |  |
|  |  |  |  |  |  | 1 | | | 2 | | | 3 | | |  |  |
| Common Factor | | | | | | 0.843 | | | 0.116 | | | 0.110 | | |  |  |
| Walking forward on the balance beam | | | | | | 0.793 | | | 0.066 | | | 0.101 | | |  |  |
| Turn around and jump | | | | | | 0.668 | | | 0.250 | | | 0.109 | | |  |  |
| Single and double foot alternating jumping grid | | | | | | 0.219 | | | 0.801 | | | 0.001 | | |  |  |
| Jump through hoops on one leg | | | | | | 0.095 | | | 0.733 | | | 0.003 | | |  |  |
| Crawling with hands and feet | | | | | | 0.169 | | | 0.693 | | | -0.028 | | |  |  |
| Sideslip step | | | | | | -0.029 | | | 0.688 | | | 0.172 | | |  |  |
| Throwing sandbags | | | | | | 0.359 | | | -0.248 | | | 0.728 | | |  |  |
| Spot kick | | | | | | -0.160 | | | 0.175 | | | 0.688 | | |  |  |
| Catch sandbags with both hands | | | | | | 0.428 | | | 0.081 | | | 0.683 | | |  |  |
| Step by step walking | | | | | | 0.474 | | | 0.098 | | | 0.482 | | |  |  |
| **Supplementary Material 10**  Table S10 KMO Value and Bartlett's Sphericity Test Results | | | | | | | | | | | | |  |  |  |  |
| Inspection items | | | Inspection indicators | | | | Statistical value | | | | | |  |  |  |  |
| KMO | | |  | | | | 0.675 | | | | | |  |  |  |  |
| Bartlett's Sphericity Test | | | Chi-square value | | | | 291.927 | | | | | |  |  |  |  |
|  | | | Degree of freedom, | | | | 45 | | | | | |  |  |  |  |
|  | | | Statistical significance | | | | 0.000 | | | | | |  |  |  |  |

| **Supplementary Material 11**  Table S11 Test Methods and Precautions | | |
| --- | --- | --- |
| Test process | Test methods | Precautions |
| Starting point | The subjects stand at the starting point, and when they hear 'the test begins,' they step onto the balance beam. | Please ensure the protection of the subjects by arranging personnel accordingly. |
| Walking forward on the balance beam | The subject stands on the balance beam, facing towards the endpoint, arms held horizontally to the sides. They actively move towards the other end of the balance beam, and the test concludes when either foot surpasses the endpoint, 3 meters from the starting point. | If the test subject lands midway, they must retry and personnel must be arranged to protect the test subject. |
| Single and double feet alternating jumping grid | The subject stands with both feet together behind the starting line, then hops into one agility ring with one foot, followed by hopping into two rings with both feet simultaneously. This sequence repeats for a total of six sets of rings, completing the test. | If the test subject steps out of the agility circle or makes any other mistake, the test will be restarted. |
| Catch sandbags with both hands | The subject stands naturally in the designated area, facing the sandbag throwing point, and catches the sandbag thrown by the tester with both hands once. The subject has a total of 3 chances, and if they catch the sandbag at least once or miss all three times, the test will end. | If the participant’s body extends beyond the square area while catching the sandbag, it will not be counted as a score. |
| Jump through hoops on one leg | The subject places their feet together, standing behind the starting line. They then swing both arms, jump off one foot, and land within an agility circle with each jump. The test concludes after successfully jumping through six circles in succession. | If the test subject steps out of the agility circle or makes any other mistake, the test will be restarted. |
| Spot kick | The subject faces towards the goal with their body and kicks the ball into the goal from a distance of 5 meters from the starting point using any part of their left (right) foot, they have 3 attempts in total, if they score once or fail to score all 3 times, the test ends similarly. | Attention: Participants must not step on or cross over the kicking line. |
| Sideslip step | The subject stands with feet apart, arms held out to the sides, left shoulder (right shoulder) facing towards the endpoint, then steps towards the endpoint, with the test concluding when either foot crosses the endpoint line. | Caution: Remind the subjects to slide along the line as much as possible. |
| Throwing sandbags | The subject stands facing the direction of the throw, with feet apart, and holds the sandbag in the left (right) hand above the head. Then, the sandbag is thrown into the circle 3 meters from the throwing line once. The subject has 3 chances, and if they hit the target once or miss all 3 times, the test ends. | Attention: Participants must not step on or cross the throwing line. |
| Crawling with hands and feet | The subject faces the direction of the endpoint, with both hands and feet touching the ground, head raised, and proceeds to crawl forward alternately with hands and feet until reaching the endpoint 6 meters from the starting point. The test concludes if the head surpasses the endpoint line. | If the examinee crawls using other parts of the body or makes any other mistakes, the test for that item should be retaken. |
| Turn around and jump | During the test, the subjects stand with their hands on their hips, feet naturally apart, and squat down naturally, then rotate and jump to the other designated direction three times (making a 180-degree turn). | Count if the subject completes within the square area; otherwise, do not count. |
| Sideward roll | The subject lies supine, with arms raised overhead and legs together, using the force generated from the shoulders and hips to roll the body towards the endpoint three times. | Ensure personnel are arranged to protect the subjects. |
| End point | After completing the straight body roll, the subject stands up and touches the yellow marked bucket, signaling the conclusion of the entire test. | Attention to the temporary dizziness that may occur after the supine roll maneuver in subjects. |
